# Supplementary material for: Iterative improvement in the automatic modular design of robot swarms
Source: PeerJ Comput Sci. 2020 Dec 7;6:e322. doi: 10.7717/peerj-cs.322 (PMC7924708; doi:10.7717/peerj-cs.322)
Supplement: Supplemental Information 3 [file peerj-cs-06-322-s003.zip › argos3/doc/api/standalone/a00529_source.html]

ARGoS: plugins/robots/generic/simulator/ground\_rotzonly\_sensor.h Source File


- Main Page
- Related Pages
- Namespaces
- Classes
- Files

- File List
- File Members

# plugins/robots/generic/simulator/ground\_rotzonly\_sensor.h

Go to the documentation of this file.

```
00001 
00007 #ifndef GROUND_ROTZONLY_SENSOR_H
00008 #define GROUND_ROTZONLY_SENSOR_H
00009 
00010 #include <string>
00011 #include <map>
00012 
00013 namespace argos {
00014    class CGroundRotZOnlySensor;
00015    class CGroundSensorEquippedEntity;
00016    class CFloorEntity;
00017 }
00018 
00019 #include <argos3/plugins/robots/generic/control_interface/ci_ground_sensor.h>
00020 #include <argos3/core/utility/math/range.h>
00021 #include <argos3/core/utility/math/rng.h>
00022 #include <argos3/core/simulator/space/space.h>
00023 #include <argos3/core/simulator/sensor.h>
00024 
00025 namespace argos {
00026 
00027    class CGroundRotZOnlySensor : public CSimulatedSensor,
00028                                  public CCI_GroundSensor {
00029 
00030    public:
00031 
00032       CGroundRotZOnlySensor();
00033 
00034       virtual ~CGroundRotZOnlySensor() {}
00035 
00036       virtual void SetRobot(CComposableEntity& c_entity);
00037 
00038       virtual void Init(TConfigurationNode& t_tree);
00039 
00040       virtual void Update();
00041 
00042       virtual void Reset();
00043 
00044    protected:
00045 
00047       CEmbodiedEntity* m_pcEmbodiedEntity;
00048 
00050       CFloorEntity* m_pcFloorEntity;
00051 
00053       CGroundSensorEquippedEntity* m_pcGroundSensorEntity;
00054 
00056       CRandom::CRNG* m_pcRNG;
00057 
00059       bool m_bAddNoise;
00060 
00062       CRange<Real> m_cNoiseRange;
00063 
00065       CSpace& m_cSpace;
00066    };
00067 
00068 }
00069 
00070 #endif
```

---

Generated on 10 Jul 2018 for ARGoS by 
 1.6.1
